# Burden of Insomnia Among Individuals With Major Depressive Disorder: Cross-Sectional Observational Study

**DOI:** 10.2196/93231

**Published:** 2026-09-21

**Authors:** Josh Hamilton, Emilie Pain, Jesper Riise, Zhiheng Zhang

**Affiliations:** 1 The Hamilton Group Behavioral Health LLC, Las Vegas, NV, United States; 2 Carenity, 1 Rue de Stockholm, Paris, 75008, France; 3 Janssen Cilag A/S, Birkerød, Denmark; 4 Janssen Scientific Affairs, Titusville, NJ, United States

**Keywords:** cross-sectional study, online study, health-related quality of life, depressive disorder, insomnia

## Abstract

**Background:**

Insomnia is a common and persistent symptom in major depressive disorder (MDD), associated with poorer outcomes and reduced quality of life (QoL). Despite its prevalence, recent patient-reported data on the burden of insomnia symptoms in MDD remains limited.

**Objective:**

This study aimed to assess the burden of insomnia among individuals with MDD experiencing insomnia symptoms (MDDIS) and its impact on patient-perceived disease burden and QoL.

**Methods:**

A noninterventional, cross-sectional online study was conducted from October 2022 to February 2023 in adults with self-reported MDD and insomnia symptoms, residing in the United States or 1 of 5 European countries (EU5: France, Germany, Italy, Spain, and the United Kingdom). Participants completed validated questionnaires (Patient Health Questionnaire-9 [PHQ-9] for depression severity and Insomnia Severity Index [ISI] for insomnia severity) and provided data on demographics, clinical history, treatment experience, and perceptions. Descriptive statistics were used to summarize results, and subgroup analyses were conducted based on depression status and severity.

**Results:**

Among 1250 participants (United States: 500; EU5: 750), most were cisgender women (United States: 363/500, 72%; EU5: 637/750, 85%), aged ~48 years, and on average 13.4 years in the United States and 11.2 years in EU5 had passed since being diagnosed with MDD. Around 60% (United States: 312/500; EU5: 439/750) reported having moderate to severe insomnia symptoms. Most had used antidepressants (United States: 460/500, 92%; EU5: 646/750, 86%) at least 40% (United States: 198/500; EU5: 343/750) in both regions reported using sleep-related medications (over-the-counter sleep aids, dual-purpose medication, or sleep-specific medication). However, insomnia symptoms persisted, with around half of participants reporting 8 to 15 insomnia nights over 2 weeks. Insomnia symptom severity differed significantly according to depression severity and current depressive episode status. Nearly all participants reported negative consequences for daily functioning, including irritability, cognitive impairment, and reduced productivity, and approximately two-thirds identified sleep disturbances as having a major impact on their daily lives. Satisfaction with current treatments, particularly their effectiveness on sleep, was low, and 65%‐71% (488/750-354/500) of participants expressed a desire for alternative therapies.

**Conclusions:**

Insomnia symptoms are highly prevalent and burdensome among patients with MDD, severely affecting daily life and perceived treatment success. Insomnia remains inadequately controlled despite high medication use. These findings emphasize the need for integrated treatment strategies targeting both mood and sleep symptoms. Given its role in depression relapse, suicidality, and treatment resistance, addressing insomnia symptoms in MDD should be a clinical priority.

## Introduction

### Background

Major depressive disorder (MDD) or major depression is the most commonly diagnosed form of depression [1] and was ranked by the World Health Organization as the third leading cause of disease burden in 2018, with projections placing it first by 2030 [2]. MDD is defined by a persistently low mood, loss of interest or pleasure (anhedonia), recurrent suicidal thoughts, and a range of physical and cognitive symptoms—including sleep disturbance, changes in appetite and weight, fatigue, psychomotor agitation or retardation, feelings of worthlessness, and difficulty concentrating [3-5]. According to the *Diagnostic and Statistical Manual of Mental Disorders* (Fifth Edition; *DSM-5*), diagnosis requires at least 5 of these symptoms lasting a minimum of 2 weeks [6].

The global incidence of MDD has steadily increased over the past 3 decades [7], with a sharp 27.6% rise in 2020 linked to the COVID-19 pandemic [8]. The most recent Global Burden of Disease (GBD) study estimated that 229 million people worldwide were affected by MDD in 2021, with the highest prevalence rate in the United States (4527 cases per 100,000 population; 15 million cases) and Europe (3771 cases per 100,000 population; 32 million cases) [7,9]. Globally, MDD is more prevalent in women, with a female-to-male ratio of approximately 1.6, as reported in the most recent GBD study, rising to 1.9 in the United States and Europe [7].

MDD severely impacts quality of life (QoL) not only due to its core symptoms but also because of functional limitations, social disruption, and comorbidities [3,10]. The severity of depressive symptoms directly correlates with greater QoL impairment [11-13]. Among these symptoms, sleep disturbances, especially insomnia, are among the most common, affecting around 60% of people with depression [14,15]. Insomnia symptoms are frequently reported as the main complaint [16], further deteriorating QoL by worsening energy levels and mood, and overall functioning [15,17]. Sleep disturbances in MDD are also linked to more severe symptoms—persistent depression, higher relapse risk, poorer response to traditional depression treatment, and increased risk of suicidality [18-26]. Insomnia symptoms often persist despite antidepressant treatment [27] and after remission of depressive symptoms, making individuals with MDD experiencing insomnia symptoms (MDDIS) particularly difficult to treat [28,29].

Though the literature suggests MDD and sleep disturbances are associated [16,20,30], the persistence and clinical consequences of insomnia symptoms highlight a critical need for interventions to address sleep disorders in MDDIS to improve patients’ outcomes. Despite extensive research [31,32], recent data capturing patient-reported outcomes on insomnia symptoms in MDD remain limited, particularly regarding how patients perceive its impact on their lives and how this knowledge can guide the development of more effective care strategies.

### Objective

To address the absence of data on patients’ perspectives regarding the impact of insomnia symptoms on patients’ lives and treatment needs, we collected current patient-reported data on insomnia symptoms among individuals with MDDIS in the United States and Europe. The primary objective of this descriptive study was to assess the burden of insomnia symptoms and patient-reported QoL in this population. We also sought to understand how patients perceive the relationship between their sleep disturbances and depression, how they perceive their current MDDIS treatments, and if they discuss their insomnia symptoms with a clinician or another member of their care team.

In this paper, we present findings from this observational study that provides insights into the lived experience of MDDIS, focusing on how insomnia symptoms shape their daily functioning and well-being.

## Methods

### Study Design

This was a noninterventional, observational, and cross-sectional web-based study targeting patients who self-reported a diagnosis of depression and symptoms of insomnia.

### Study Population

Eligible participants were adults (≥18 years) residing in the United States, or in 1 of 5 European countries (EU5; France, Germany, Italy, Spain, or the United Kingdom), who self-reported a health care professional diagnosis of MDD, self-reported insomnia symptoms, and who provided informed consent. Participants who did not meet the inclusion criteria or who did not fully complete the questionnaire were excluded from the analysis. No medical records, prescriptions, or physician confirmation were requested as part of the study.

The target sample size was 1250 participants: 500 from the United States and 750 from EU5.

Participants were recruited through Carenity (ELSE CARE), an online community launched in 2011, where both patients and caregivers share experiences, access health-related information, and participate in online research studies. Carenity members were invited to participate by email. Recruitment also involved online campaigns (Facebook; Meta Platforms Inc. and Google; Alphabet Inc.). No incentives were provided for participation in the study.

Data were collected between October 2022 and February 2023.

### Ethical Considerations

The study protocol was approved by the Western Institutional Review Board–Copernicus Group (WCG IRB; approval number 20223963). All participants provided electronic informed consent prior to completing the study.

### Data Collection and Measures

#### Overview

Participants completed a self-administered online questionnaire. The study included 39 closed-ended questions covering participants’ demographic characteristics, clinical profiles, and the impact of MDDIS on daily life. All questions were mandatory, and the study required approximately 15 minutes to complete. Study-specific questions were translated by a certified translation agency in the native language of each targeted country. The translated versions were proofread by native speakers for consistency. The Patient Health Questionnaire (PHQ-9) and the Insomnia Severity Index (ISI) questionnaires were administered using the official translated versions provided by the respective copyright holders.

#### Sociodemographic Variables

Participants reported their sex, year of birth, and country of residence. Sex was categorized into 5 groups: cisgender male, cisgender female, transgender male, transgender female, and nonbinary.

#### Clinical Variables

Participants self-reported a health care professional–confirmed diagnosis of MDD and provided information on the date of diagnosis, the type of health care professional having diagnosed MDD, their comorbidities, MDD characteristics, current and past depression treatments, insomnia symptoms characteristics, and sleep treatment history.

The PHQ-9, a 9-item patient-reported instrument for assessing depression severity, was administered using validated language versions that have demonstrated good psychometric properties [33-37]. The total score ranges from 0 to 27, with higher scores indicating greater depression severity. Severity is categorized as: minimal (0-4), mild (5-9), moderate (10-14), moderately severe (15-19), and severe (20-27).

#### Outcome Variables

In the questionnaire, participants were informed that “Insomnia, or trouble sleeping, can include falling asleep, staying asleep or waking up early, and being unable to go back to sleep.”

To evaluate the burden of insomnia symptoms in MDDIS, participants were asked how many nights they had experienced insomnia symptoms or used treatments for them in the 2 weeks prior to study inclusion (for those with a current depressive episode) or prior to their most recent episode. These are referred to as “insomnia nights” in the patient questionnaire and the paper. Participants also reported the duration of their insomnia symptoms or treatment use. To measure nocturnal and daytime symptoms of insomnia and assess its severity, the ISI, a validated 7-item scale, was also administered [38]. The total score ranges from 0 to 28, with higher scores indicating greater insomnia symptom severity. Insomnia symptoms severity is categorized as: no insomnia symptoms (0-7), subthreshold (8-14), moderate (15-21), and severe (22-28).

To evaluate the impact of MDDIS on patients’ QoL, participants were asked to report, through 2 closed-ended self-designed questions, the MDDIS symptoms that had the greatest impact on their global daily life, and the consequences of sleep disruptions on their daily life during the most recent episode.

Participants were also asked to report their perceptions of the relationship between their MDD and insomnia symptoms, their satisfaction with treatments for MDD and insomnia symptoms, and their unmet needs regarding these treatments.

### Statistical Analysis

The study population included all participants who met the study selection criteria and completed the questionnaire. Descriptive statistics were used to characterize the study population and summarize study responses. Quantitative data are reported using mean and its 95% CI and qualitative data are reported as frequencies and percentages. Results are presented separately by region of residence (United States or EU5). Descriptive subgroup analyses were conducted according to current depressive episode status, depression severity, and other relevant clinical characteristics. Differences in the distribution of categorical variables between subgroups were assessed using Pearson chi-square tests or Fisher exact test. All statistical tests were 2-sided, and a *P* value <.05 was considered statistically significant.

All data management and statistical analysis were performed using R software (version 4.1.2; R Core Team).

## Results

### Study Population Characteristics

**Figure 1.**
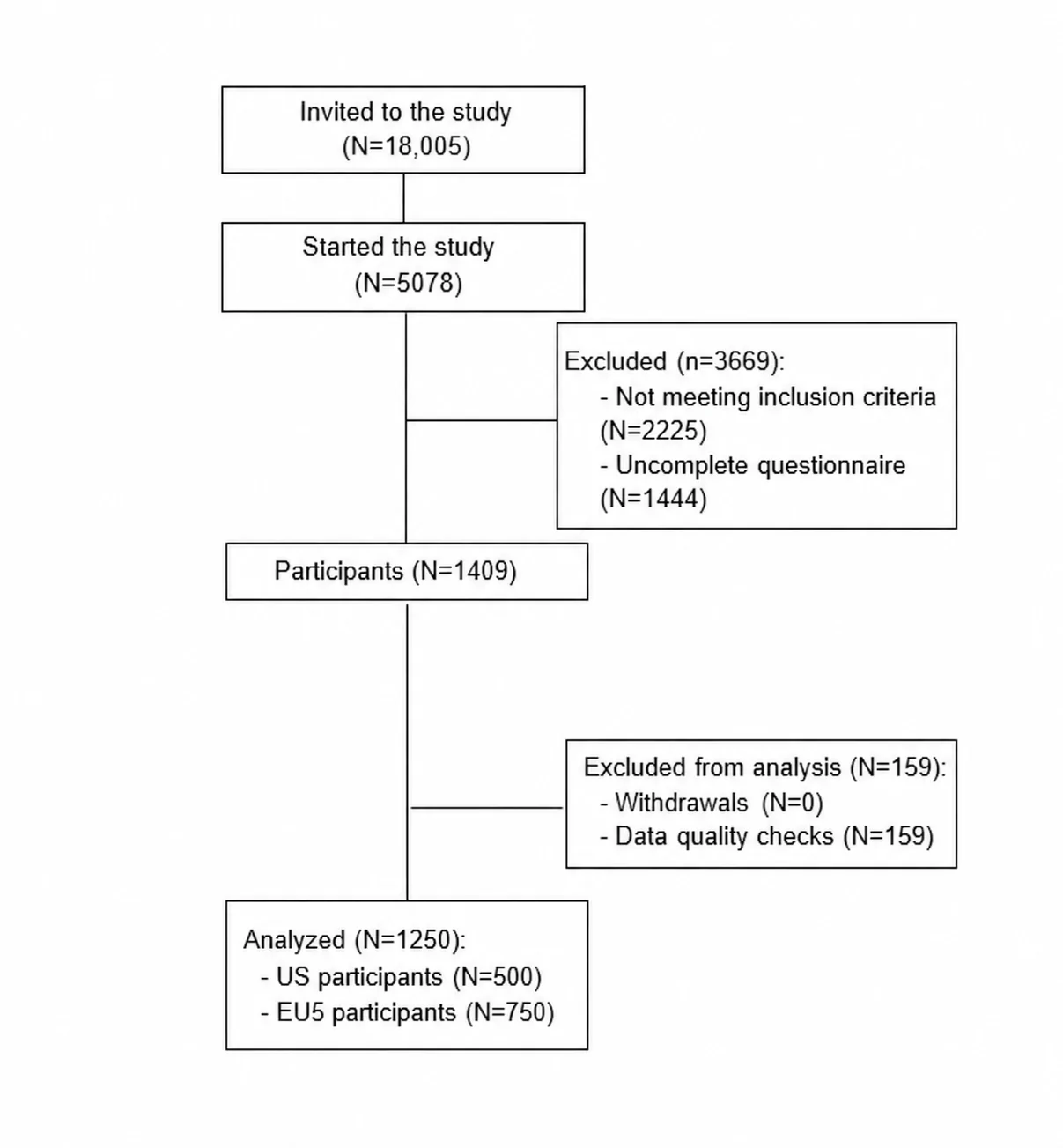
Flowchart of the study population. US: United States; EU5: France, Germany, Italy, Spain, and the United Kingdom.

Recruitment targets were met in both regions (United States and EU5), resulting in a total of 1250 participants: 500 in the United States and 750 in EU5. Details of participant disposition are shown in Figure 1. Demographic and clinical characteristics of the study population are detailed in Table 1.

In both the United States and EU5, the majority of participants were cisgender women (United States: 363/500, 72%, and EU5: 637/750, 85%). A total of 12% (62/500) of US participants identified as transgender or nonbinary, compared to 3% (21/750) in the EU5 sample. Mean age at inclusion was similar across regions (United States: 48.1, 95% CI 46.5‐49.7 years; EU5: 47.5, 95% CI 46.5‐48.6 years) and most participants were diagnosed with MDD in their mid-thirties. In both regions, the majority of participants reported receiving their MDD diagnosis from a psychiatrist, with 79% (127/161) in Italy, 72% (111/154) in Germany, 68% (95/140) in Spain, 59% (98/167) in France, and 28% (36/128) in the United Kingdom (Table S1 in Multimedia Appendix 1).

**Table 1.** Demographic and health characteristics of the study population.

Participants characteristics	US participants (n=500), n (%)	EU5^a^ participants (n=750), n (%)
Demographic variables		
Sex		
Cisgender male	68 (14)	68 (9)
Cisgender female	363 (72)	637 (85)
Transgender male	24 (5)	6 (1)
Transgender female	3 (1)	0 (0)
Nonbinary	35 (7)	15 (2)
Prefer not to answer	7 (1)	24 (3)
Age (years), mean (95% CI)	48.1 (46.5-49.7)	47.5 (46.5-48.6)
Age groups (years), n (%)		
18‐30	127 (25)	123 (16)
31‐60	217 (43)	499 (67)
>60	156 (31)	128 (17)
Disease variables		
Time since MDD^b^ diagnosis (years), mean (95% CI)	13.4 (12.2-14.5)	11.2 (10.4-12.0)
Time since MDD diagnosis (years), n (%)		
0‐5	201 (40)	338 (45)
6‐20	153 (31)	244 (33)
>20 year	146 (29)	168 (22)
Age at MDD diagnosis (years), mean (95% CI)	34.7 (33.2‐36.2)	36.3 (35.3‐37.4)
Age at MDD diagnosis (years), n (%)		
≤18	98 (20)	90 (12)
19‐30	128 (26)	207 (28)
31‐60	229 (46)	415 (55)
>60	45 (9)	38 (5)
HCPs^c^ who diagnosed MDD, n (%)		
Psychiatrist	292 (58)	467 (62)
General practitioner	183 (37)	237 (32)
Others^d^	25 (5)	46 (6)
Mental health comorbidities, n (%)		
Generalized anxiety disorder	306 (61)	372 (50)
Chronic anxiety (stress)	242 (48)	376 (50)
Posttraumatic stress disorder	202 (40)	181 (25)
Social anxiety disorder	191 (38)	233 (31)
Panic disorder (or panic attacks)	179 (36)	256 (34)
Attention deficit disorder with or without hyperactivity	98 (19)	40 (6)
Eating disorder	75 (15)	164 (22)
Any personality disorder	56 (11)	109 (14)
Obsessive compulsive disorder	67 (13)	61 (9)
Others	55 (11)	109 (14)
None	41 (8)	57 (9)
Current depressive episode, n (%)		
Yes	259 (52)	448 (60)
Not sure but depression symptoms	170 (34)	207 (28)
Time elapsed since the current depressive episode^e^ (years), n (%)		
≤1	240 (56)	374 (57)
1-5	97 (23)	155 (24)
>5	92 (21)	122 (19)
Unknown	0 (0)	4 (1)
No	71 (14)	95 (13)
Time elapsed since the most recent depressive episode (years), n (%)		
≤1	56 (79)	52 (55)
1-5	10 (14)	22 (23)
>5	5 (7)	21 (22)
Duration of the most recent depressive episode, n (%)		
≤6 months	35 (49)	39 (41)
6-12 months	19 (27)	31 (33)
>1 year	17 (24)	25 (27)

a EU5: France, Germany, Italy, Spain, and the United Kingdom.

b MDD: major depressive disorder.

c HCP: health care practitioner.

d “Others” includes psychologist, therapist, mental health service, mental health counselor, nurse practitioner, neurologist, counseling center, not specified.

e Time elapsed since the current episode applies to participants with current MDD and those who reported experiencing symptoms of depression.

Comorbid mental health conditions were prevalent. The majority of participants from both regions reported at least one additional condition, most commonly generalized anxiety disorder, chronic anxiety or stress, posttraumatic stress disorder, social anxiety disorder, and panic disorder. At the time of the study, 86% (429/500) of United States and 87% (655/750) of EU5 participants were experiencing depressive symptoms or were in an active depressive episode, which had started less than a year earlier for over half of the participants. Among those not in an active episode, most had experienced one within the past year.

#### Depression Severity

**Figure 2.**
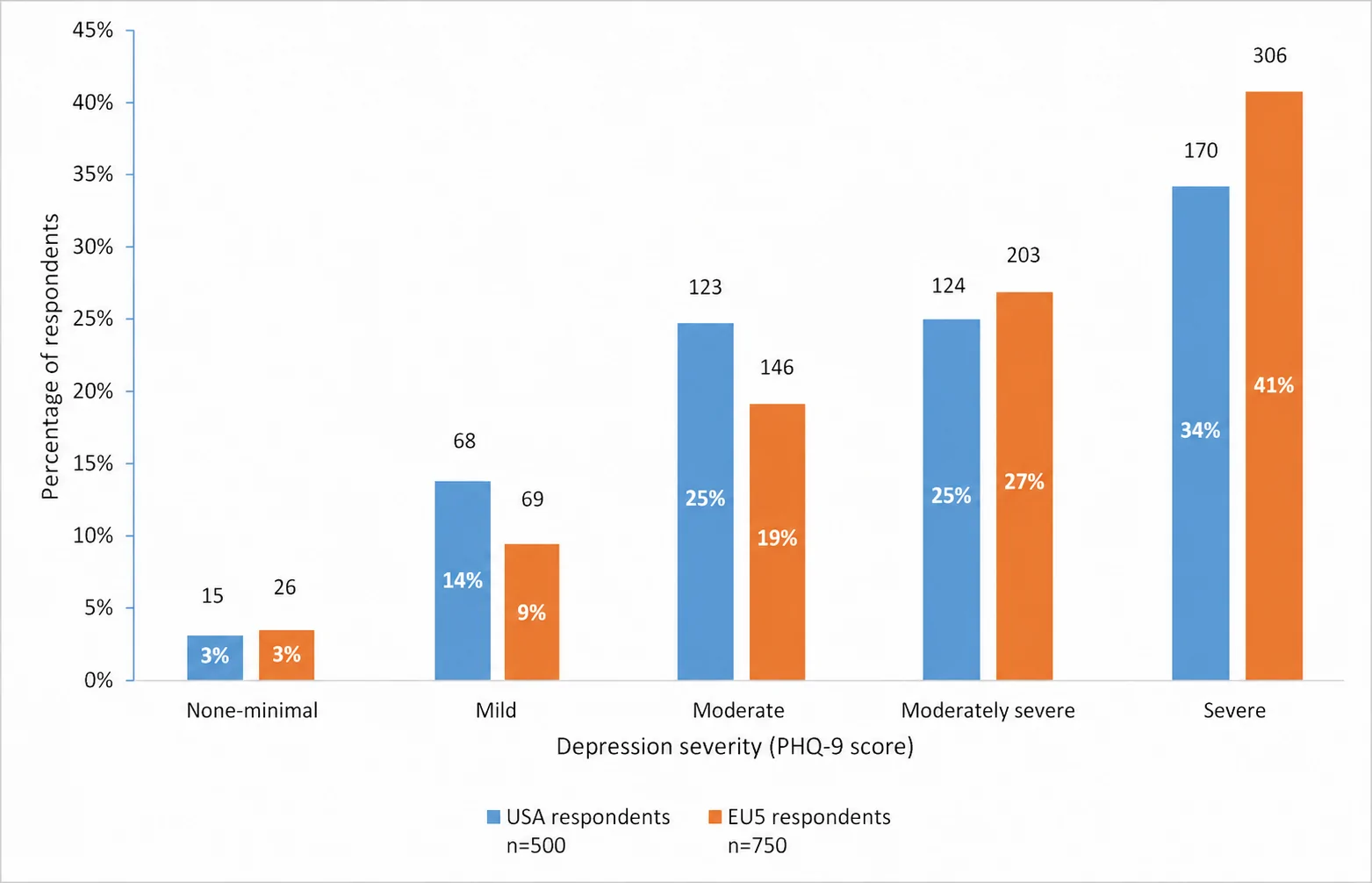
Severity of depression in US (n=500) and EU5 (n=750) participants as measured by the Patient Health Questionnaire-9. EU5: France, Germany, Italy, Spain, and the United Kingdom; PHQ-9: Patient Health Questionnaire-9.

As measured by the PHQ-9, 34% (170/500) of US participants were classified as having severe depression, 25% (124/500) as moderately severe, and 25% (123/500) as moderate depression (Figure 2). In the EU5 region, 41% (306/750) of participants had severe depression, 27% (203/750) moderately severe, and 19% (146/750) moderate depression. Descriptively, the proportion of participants with severe depression was highest in Spain, the United Kingdom, and Germany, and lower in France and Italy (Table S1 in Multimedia Appendix 1). Across both regions, the distribution of depression severity differed according to age group (both *P*<.001) and the number of insomnia nights reported during the previous 2 weeks (United States *P*<.001; EU5 *P*=.04), with a higher proportion of younger participants (18-30 years) and those who had experienced more insomnia nights reporting severe depression (Tables 2 and 3). Among US participants, a greater proportion of transgender or nonbinary participants had severe depression (*P*=.02).

**Table 2.** Depression severity by sex, age, and insomnia symptoms frequency in US participants (n=500) as measured by the PHQ-9^a^.

	None-minimal (n=15)	Mild (n=68)	Moderate (n=123)	Moderately severe (n=124)	Severe (n=170)	*P* values
Sex, n (%)						.02^b^
Cisgender female	13 (4)	54 (15)	91 (25)	92 (25)	113 (31)	
Cisgender male	2 (3)	10 (15)	17 (25)	20 (29)	19 (28)	
Transgender male and female and nonbinary	0 (0)	4 (6)	15 (22)	12 (17)	38 (55)	
Age groups (years), n (%)						<.001
18‐30	2 (2)	8 (6)	23 (18)	27 (21)	67 (53)	
31‐60	8 (4)	37 (17)	44 (20)	58 (27)	70 (32)	
>60	5 (3)	23 (15)	56 (36)	39 (25)	33 (21)	
Insomnia nights^c^, n (%)						<.001
0‐7	8 (4)	37 (20)	49 (26)	32 (17)	62 (33)	
8‐15	2 (1)	6 (6)	30 (18)	56 (34)	68 (41)	

a PHQ-9: Patient Health Questionnaire-9.

b Fisher exact test.

c Insomnia nights are defined here as nights with insomnia symptoms or taking treatments for insomnia symptoms (for n=276 for 8‐15 insomnia nights; for the United States, n=188 for 0‐7 insomnia nights and n=166 for 8‐15 insomnia nights).

**Table 3.** Depression severity by sex, age, and insomnia symptoms frequency in EU5^a^ (N=750) participants as measured by the PHQ-9^b^.

	None-minimal (n=26)	Mild (n=69)	Moderate (n=146)	Moderately severe (n=203)	Severe (n=306)	*P* values
Sex, n (%)						.45^c^
Cisgender female	21 (3)	60 (9)	122 (19)	175 (27)	259 (42)	
Cisgender male	5 (7)	7 (10)	16 (24)	16 (24)	24 (35)	
Transgender male and female and nonbinary	0 (0)	2 (4)	8 (18)	12 (27)	23 (51)	
Age groups (years), n (%)						<.001
18‐30	2 (2)	2 (2)	16 (13)	32 (26)	71 (58)	
31‐60	17 (3)	47 (9)	100 (20)	137 (27)	198 (40)	
>60	7 (5)	20 (16)	30 (23)	34 (27)	37 (29)	
Insomnia nights^d^, n (%)						.04
0‐7	4 (2)	21 (10)	38 (18)	58 (28)	85 (41)	
8‐15	6 (2)	12 (4)	39 (14)	76 (28)	143 (52)	

a EU5: France, Germany, Italy, Spain, and the United Kingdom.

b PHQ-9: Patient Health Questionnaire-9.

c Fisher exact test.

d Insomnia nights are defined here as nights with insomnia symptoms or taking treatments for insomnia symptoms (for EU5, n=206 for 0‐7 insomnia nights and n=276 for 8‐15 insomnia nights; n=166 for 8‐15 insomnia nights).

#### Burden of Depressive Symptoms

**Figure 3.**
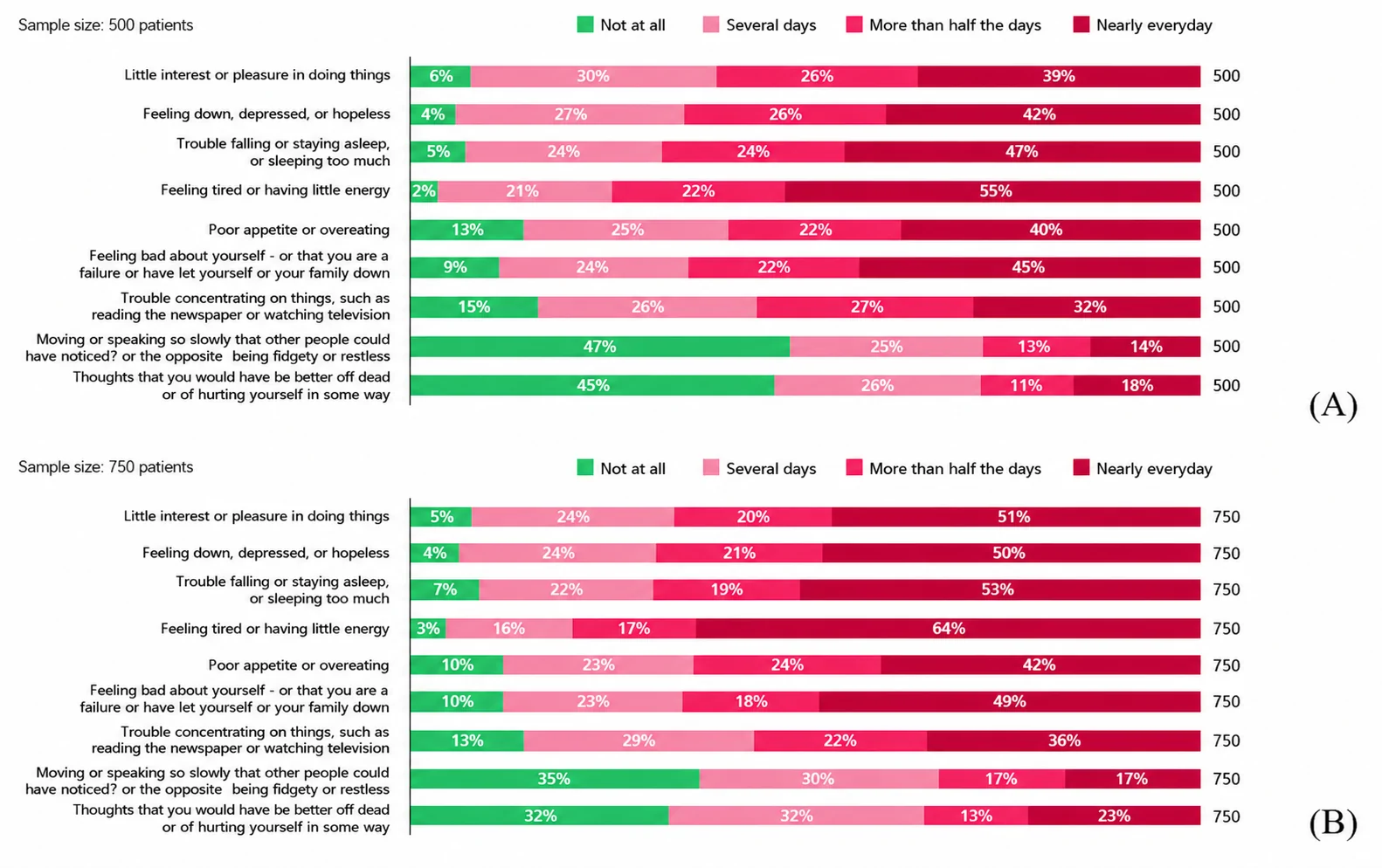
Burden of depressive symptoms in United States (A, n=500) and EU5 (B, n=750) participants, PHQ-9: Patient Health Questionnaire.

At least half of the participants considered each PHQ-9 depressive symptom burdensome. In both regions, the most frequently reported disturbing symptoms, reported by 93% or more of participants, were low energy (United States: 491/500 and EU5: 731/750), depressed mood (United States: 478/500 and EU5: 722/750), sleep issues (United States: 475/500 and EU5: 701/750), and anhedonia (United States: 471/500 and EU5: 714/750; Figures 3A and 3B). Approximately half of the participants in both regions were impacted by sleep disturbances nearly every day.

#### MDDIS Treatment

**Figure 4.**
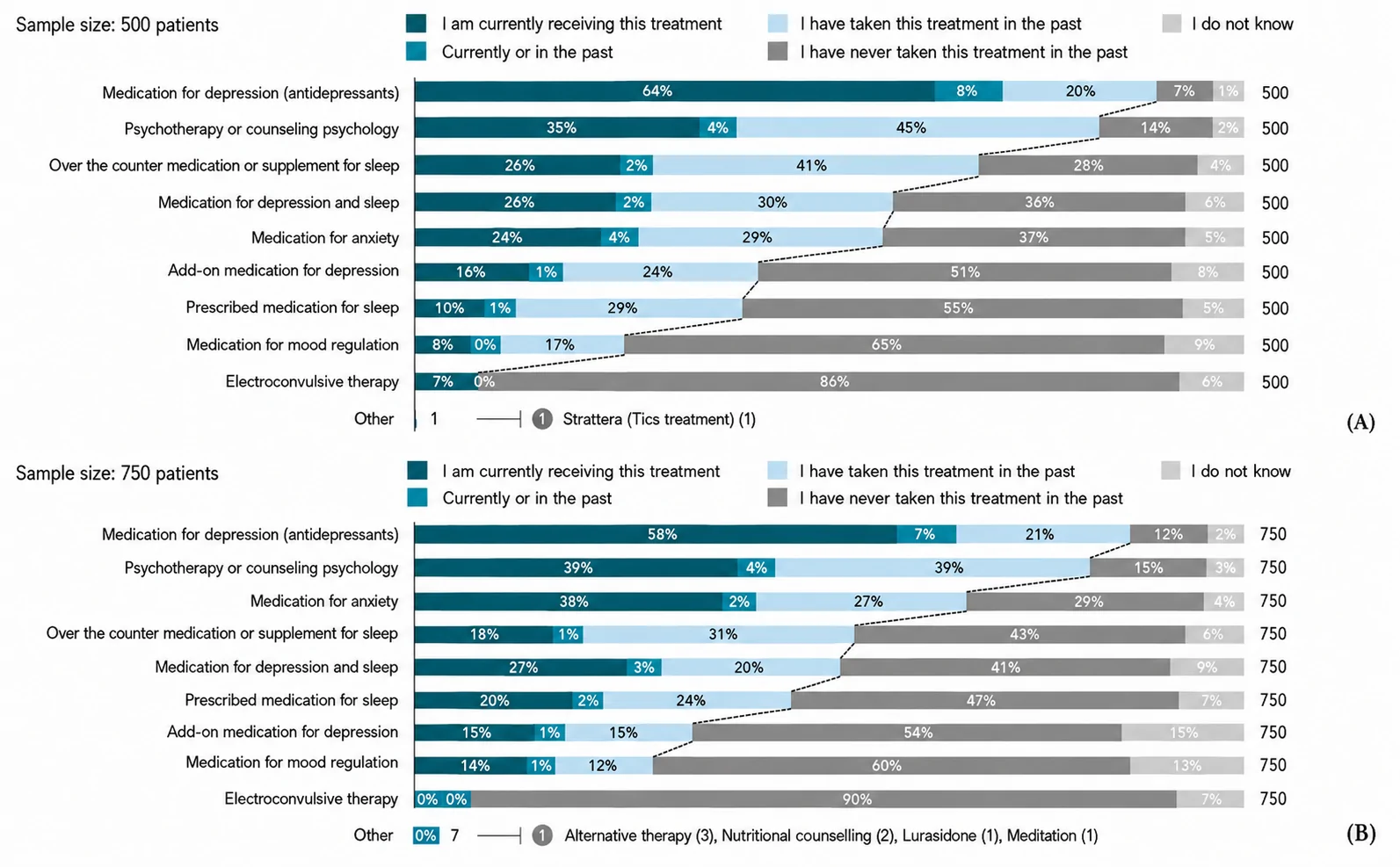
History of treatment for US (N=500) and EU5 (N=750) participants with major depressive disorder (MDD) with insomnia symptoms. EU5: France, Germany, Italy, Spain, and the United Kingdom.

Most participants had received antidepressant therapy (United States: 460/500, 92% and EU5: 646/750, 86%) and psychological care (United States: 421/500, 84% and EU5: 619/750, 82%) (Figure 4). Sleep-related treatments were also common. In the United States, 68% (341/500) reported using or having used over-the-counter (OTC) sleep aids, 58% (292/500) medications targeting both sleep and depression (dual-purpose medication), and 40% (198/500) prescribed sleep-specific medication. In the EU5, these figures were 51% (382/750), 50% (374/750), and 46% (343/750), respectively. At the time of the study, 28% (138/500 and 140/500) of US participants were taking OTC or dual-purpose medication and 11% (54/500) prescribed sleep-specific treatment. In the EU5, 19% (146/750) were using OTC sleep aids, 30% (222/750) used dual-purpose medication, and 22% (161/750) were receiving prescribed sleep-specific treatment.

During their current or most recent depressive episode, 36% (160/438) of US participants and 30% (193/643) of EU5 participants reported changing depression treatment 3 times or more.

### Burden of Insomnia Symptoms in MDDIS and Its Impact on Patient-Reported Outcomes and QoL

#### Insomnia Symptoms Frequency

**Figure 5.**
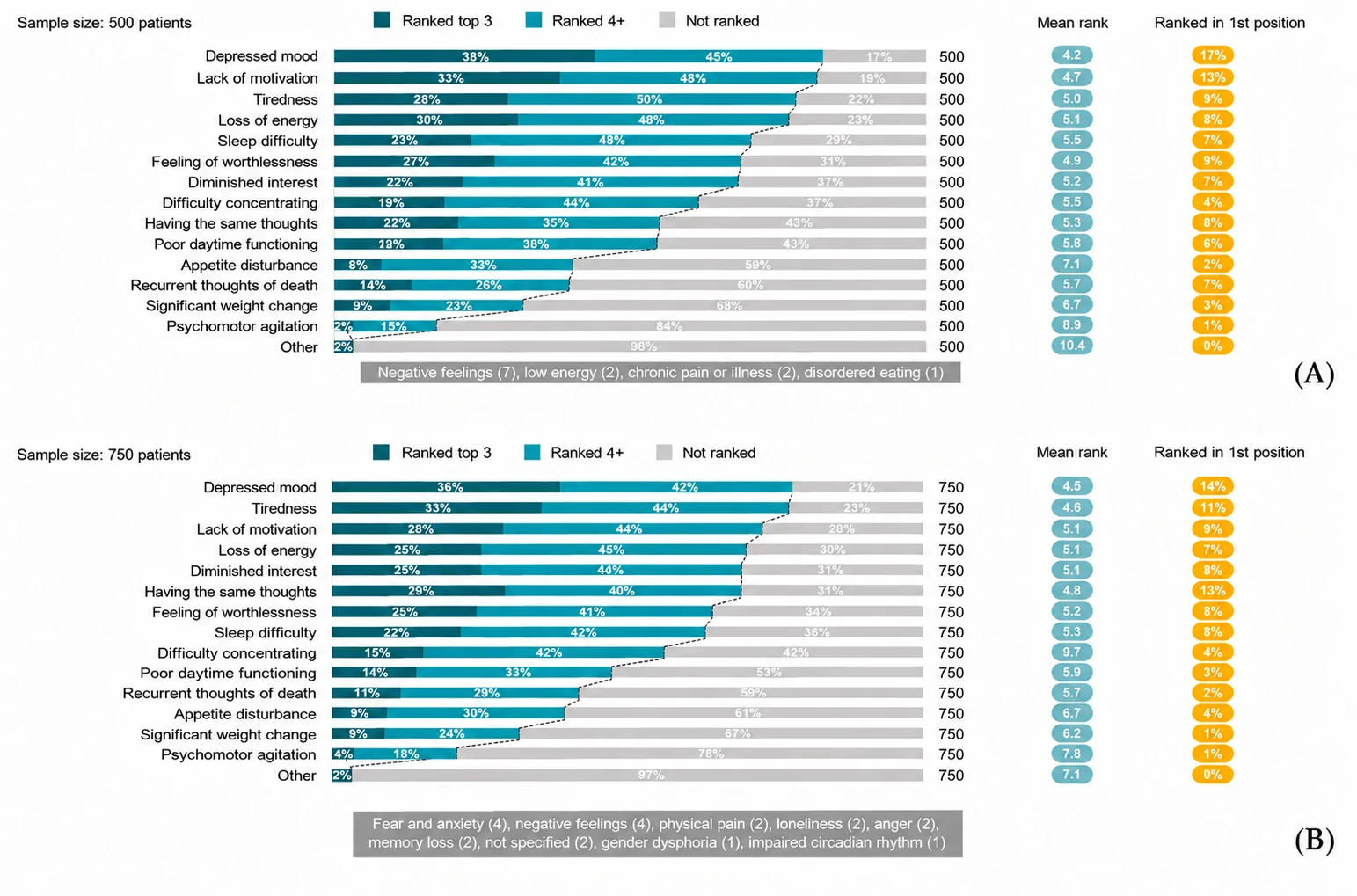
Impact of major depressive disorder with insomnia symptoms on participants’ life in the US (A, n=500) and EU5 (n=750) participants. EU5: France, Germany, Italy, Spain, and the United Kingdom.

In the US sample, participants reported a mean of 8.1 (95% CI 7.6-8.6) insomnia nights over the 2 previous weeks. Most (354/500, 71%) reported life-impacting sleep difficulties (Figure 5) and had experienced insomnia symptoms for less than 6 months (307/500, 61%; Table 4). The distribution of insomnia-night categories (0-7 vs 8-15 nights) differed according to current depressive episode status (*P*=.05) and depression severity (*P*<.001). In particular, the proportion of US participants reporting 8-15 insomnia nights was higher among those with moderately severe (56/88, 64%) or severe depression (68/130, 52%) than among those with lower depression severity categories. The distribution of insomnia-night categories also differed according to sleep medication use (*P*=.04), with a higher proportion of participants reporting 8-15 insomnia nights among those reporting using OTC or prescribed sleeping medication (153/312, 49% vs 13/42, 31% for nonsleeping medication users).

**Table 4.** Number of insomnia nights in the past 2 weeks by depression status, severity, sleep medication use, and duration of insomnia symptoms in United States (n=354) and EU5^a^ (n=482) participants.

Characteristics	US participants	EU5 participants
Number of insomnia nights^b^, mean (95% CI)	8.1 (7.6‐8.6)	9.0 (8.6‐9.4)
Number of insomnia nights, depressive episode status groups		
Currently in a depressive episode, n	310	434
0‐7 nights, n (%)	158 (51)	186 (43)
8‐15 nights, n (%)	152 (49)	248 (57)
Not currently in a depressive episode, n	44	48
0‐7 nights, n (%)	30 (68)	20 (42)
8‐15 nights, n (%)	14 (32)	28 (58)
*P* value	.047	.99
Number of insomnia nights, depression severity groups		
None-minimal, n	10	10
0‐7 nights, n (%)	8 (80)	4 (40)
8‐15 nights, n (%)	2 (20)	6 (60)
Mild, n	43	33
0‐7 nights, n (%)	37 (86)	21 (64)
8‐15 nights, n (%)	6 (14)	12 (36)
Moderate, n	79	77
0‐7 nights, n (%)	49 (62)	38 (49)
8‐15 nights, n (%)	30 (38)	39 (51)
Moderately severe, n	88	134
0‐7 nights, n (%)	32 (36)	58 (43)
8‐15 nights, n (%)	56 (64)	76 (57)
Severe, n	130	228
0‐7 nights, n (%)	62 (48)	85 (37)
8‐15 nights, n (%)	68 (52)	143 (63)
*P* value	<.001	.04
Number of insomnia nights, sleep medication groups		
Over-the-counter or prescribed sleeping medication, n	312	387
0‐7 nights, n (%)	159 (51)	160 (41)
8‐15 nights, n (%)	153 (49)	227 (59)
No sleeping medication, n	42	95
0‐7 nights, n (%)	29 (69)	46 (48)
8‐15 nights, n (%)	13 (31)	49 (52)
*P* value	.04	.26
Duration of insomnia symptoms or treatment, N	500	750
<6 months, n (%)	307 (61)	474 (63)
6 months-1 year, n (%)	97 (19)	115 (15)
>1 year, n (%)	93 (19)	160 (21)
Unknown, n (%)	3 (1)	1 (0)

a EU5: France, Germany, Italy, Spain, and the United Kingdom.

b Insomnia nights are defined as nights with insomnia symptoms or taking treatments for insomnia symptoms.

In the EU5 sample, an average of 9.0 (95% CI 8.6‐9.4) insomnia nights were reported by 482/750 (64%) participants with sleep disturbances impacting their life, and 63% (474/750) had experienced insomnia symptoms for less than 6 months (Figure 5 and Table 4). The distribution of insomnia-night categories differed across depression severity categories (*P*=.04). However, no statistically significant differences were observed according to current depressive episode status or sleep medication use. The majority of EU5 participants, including those receiving OTC or prescribed sleeping medications, continued to report 8-15 insomnia nights over the previous 2 weeks.

#### Insomnia Symptoms Severity

**Figure 6.**
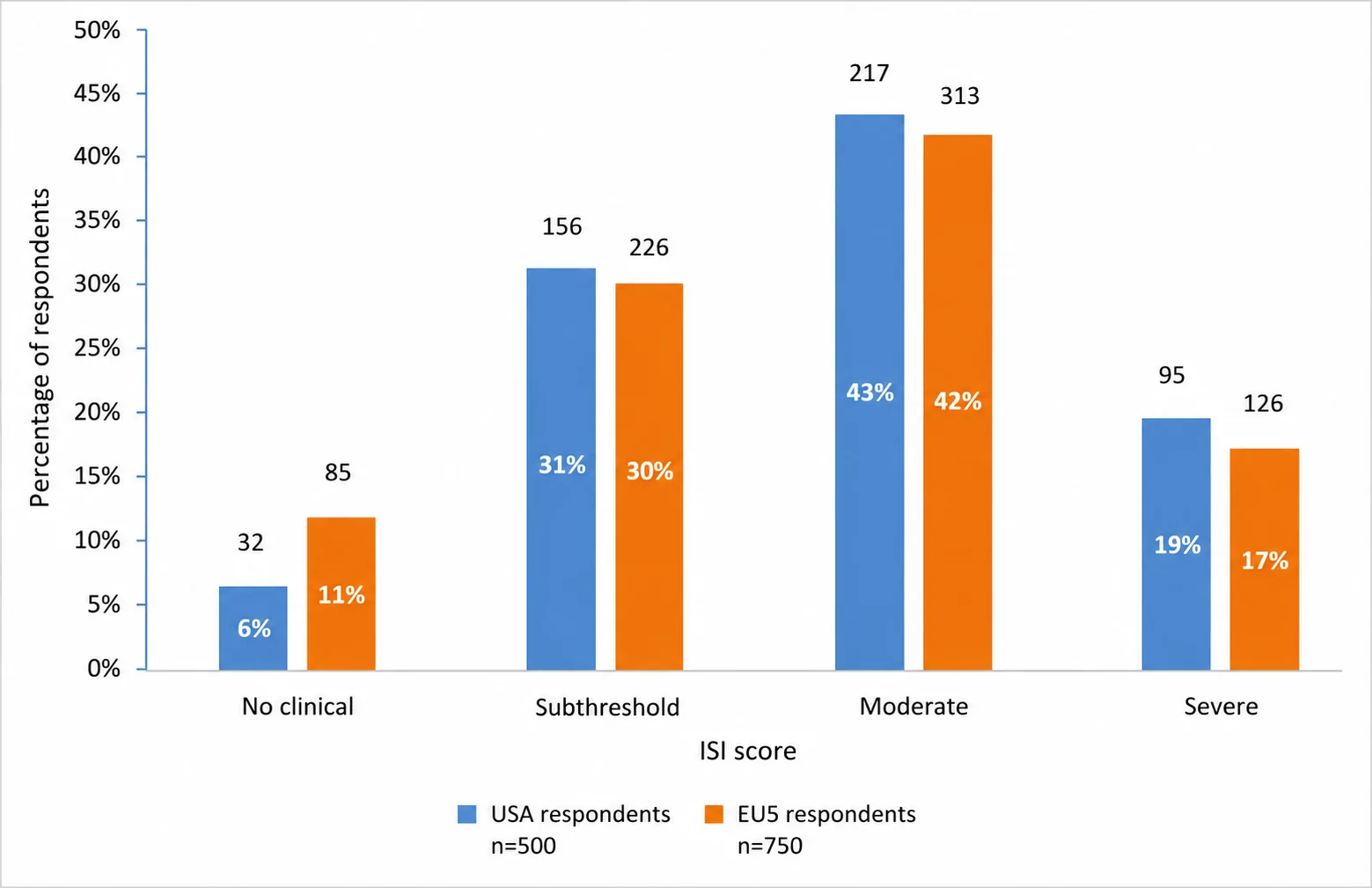
Severity of insomnia symptoms in US (n=500) and EU5 (n=750) participants as measured by the Insomnia Severity Index. EU5: France, Germany, Italy, Spain, and the United Kingdom; ISI: insomnia severity index.

Based on ISI scores, 62% (312/500) of the US and 59% (439/750) of the EU5 participants had moderate to severe insomnia symptoms (Figure 6). A total of 26% (37/140) of participants in Spain, 20% (25/128) in the United Kingdom, 18% (27/154) in Germany, 16% (26/167) in France, and 7% (11/161) in Italy reported severe insomnia symptoms (Table S1 in Multimedia Appendix 1). In both regions, the distribution of insomnia severity categories differed significantly across depression severity categories and according to current depressive episode status (all *P*<.001; Tables 5 and 6). A greater proportion of participants in the more severe depression categories were classified as having moderate or severe insomnia symptoms, with 33% (57/170) of US participants and 30% (92/306) of EU5 participants with severe depression reporting severe insomnia symptoms. Additionally, 71% of both United States (185/259) and EU5 (315/448) participants currently experiencing a depressive episode were classified as having moderate or severe insomnia symptoms. In both regions, 40% (United States: 200/500 and EU5: 300/750) of participants considered insomnia symptoms interfered with their daily functioning, and more than half (279/500, 56% of United States and 437/750, 58% of EU5 participants) believed that this impairment was noticeable to others. Dissatisfaction with sleep patterns was reported by 77% (385/500) of US participants and 68% (509/750) of those in the EU5, while 34% (167/500; United States) and 30% (219/750; EU5) expressed important distress or concern about their sleep (Table 7).

**Table 5.** Severity of insomnia symptoms by depression status and severity in US participants (n=500) as measured by the ISI^a^.

	No clinical insomnia symptoms (n=32)	Mild to moderate (n=156)	Moderate (n=217)	Severe (n=95)	*P* value
Current depression status, n (%)					<.001
Not currently in a depressive episode (n=71)	12 (17)	33 (46)	20 (28)	69 (9)	
Unsure but experiencing depression symptoms (n=170)	10 (6)	59 (35)	79 (46)	22 (13)	
Currently in a depressive episode (n=259)	10 (4)	64 (25)	118 (45)	67 (26)	
Depression severity, n (%)					<.001^b^
None or minimal (n=15)	5 (33)	8 (53)	2 (13)	0 (0)	
Mild (n=68)	9 (13)	34 (50)	21 (31)	4 (6)	
Moderate (n=123)	9 (7)	48 (39)	56 (45)	10 (8)	
Moderately severe (n=124)	7 (6)	31 (25)	62 (50)	24 (19)	
Severe (n=170)	2 (1)	35 (20)	76 (45)	57 (33)	

a ISI: Insomnia Severity Index.

b Fisher exact test.

**Table 6.** Severity of insomnia symptoms by depression status and severity in EU5^a^ (N=750) participants as measured by the ISI^b^.

	No clinical insomnia symptoms, n=85	Mild to moderate, n=226	Moderate, n=313	Severe, n=126	*P* values
Current depression status, n (%)					<.001
Not currently in a depressive episode (n=95)	30 (32)	36 (38)	23 (24)	6 (6)	
Unsure but experiencing depression symptoms (n=207)	31 (15)	81 (39)	77 (37)	18 (9)	
Currently in a depressive episode (n=448)	24 (5)	109 (24)	213 (48)	102 (23)	
Depression severity, n (%)					<.001
None or minimal (n=26)	18 (69)	6 (23)	1 (4)	1 (4)	
Mild (n=69)	21 (30)	33 (48)	13 (19)	2 (3)	
Moderate (n=146)	24 (16)	64 (44)	52 (36)	6 (4)	
Moderately severe (n=203)	14 (7)	60 (29)	104 (51)	25 (12)	
Severe (n=306)	8 (3)	63 (20)	143 (47)	92 (30)	

a EU5: France, Germany, Italy, Spain, and the United Kingdom.

b ISI: Insomnia Severity Index.

**Table 7.** Insomnia Severity Index.

ISI^a^ domains	US participants (n=500), n (%)	EU5^b^ participants (n=750), n (%)
Difficulty initiating sleep		
None	28 (6)	77 (10)
Mild	64 (13)	135 (18)
Moderate	202 (40)	238 (32)
Severe	135 (27)	209 (28)
Very severe	71 (14)	91 (12)
Difficulty staying asleep		
None	39 (8)	83 (11)
Mild	70 (14)	130 (17)
Moderate	172 (34)	248 (33)
Severe	139 (28)	205 (27)
Very severe	80 (16)	84 (11)
Problems waking up too early		
None	89 (18)	145 (19)
Mild	101 (20)	141 (19)
Moderate	145 (29)	202 (27)
Severe	109 (22)	168 (22)
Very severe	56 (11)	94 (13)
Satisfaction with current sleep pattern		
Very satisfied	9 (2)	14 (2)
Satisfied	19 (4)	65 (9)
Moderately satisfied	87 (17)	162 (22)
Dissatisfied	203 (41)	290 (39)
Very dissatisfied	182 (36)	219 (29)
Noticeability of impairment attributed to the sleep problem		
Not at all	15 (3)	42 (6)
A little	56 (11)	87 (12)
Somewhat	150 (30)	184 (25)
Much	173 (35)	250 (33)
Very much	106 (21)	187 (25)
Degree of distress or concern caused by the sleep problem		
Not at all	78 (16)	147 20)
A little	112 (22)	180 (24)
Somewhat	143 (29)	204 (27)
Much	104 (21)	154 (21)
Very much	63 (13)	65 (9)
Interference with daily functioning		
Not at all	27 (5)	88 (12)
A little	100 (20)	159 (21)
Somewhat	173 (35)	203 (27)
Much	111 (22)	206 (27)
Very much	89 (18)	94 (13)

a ISI: Insomnia Severity Index.

b EU5: France, Germany, Italy, Spain, and the United Kingdom.

#### Consequences of Insomnia Symptoms on Daily Functioning and QoL

Nearly all participants (489/494, 99% of United States and 692/730, 95% of EU5) reported at least one consequence of insomnia symptoms on their daily lives, with an average of 2.8 (95% CI 2.6‐2.9) in the United States and 2.5 (95% CI 2.4‐2.6) in the EU5 region. Approximately 70% (360/494, 73% in United States and 507/730, 69% in EU5) of participants reported more than one consequence, most commonly irritability, concentration difficulties, and reduced productivity (Table 8). The reported consequences of insomnia symptoms differed according to current depressive episode status for certain outcomes (Tables 8 and 9). In the US sample, participants currently experiencing a depressive episode were more likely to report reduced productivity (175/256, 68% vs 19/69, 27%; *P*<.001) and concentration difficulties (160/256, 62% vs 28/69, 40%; *P*=.004) than those not currently experiencing a depressive episode. No significant differences were observed for irritability, increased caffeine consumption, daytime sleep episodes, other consequences, or reporting no consequences. In the EU5 sample, significant differences were only observed for irritability (*P*=.006) and concentration difficulties (*P*=.02). Participants currently experiencing a depressive episode were more likely to report irritability (320/443, 72% vs 48/87, 55%) and concentration difficulties (272/443, 61% vs 40/87, 46%) than those not currently experiencing a depressive episode.

**Table 8.** Consequences of insomnia symptoms on daily life in US participants (n=494) with an ISI^a^ score >0**.** Participants were asked “What are/were the consequences of your sleep difficulty symptom(s) on your daily life during your most recent depressive episode?”

Consequences of insomnia symptoms^b^	US participants with ISI score >0				
	Not currently in a depressive episode (n=69)	Unsure but experiencing depression symptoms (n=169)	Currently in a depressive episode (n=256)	Total (n=494)	*P* values
Irritability, n (%)	44 (64)	132 (78)	182 (71)	358 (72)	.06
Reduced productivity, n (%)	19 (27)	103 (61)	175 (68)	297 (60)	<.001
Concentration difficulties, n (%)	28 (40)	106 (63)	160 (62)	294 (60)	.004
Increased caffeine consumption, n (%)	26 (38)	91 (54)	121 (47	238 (48)	.07
Daytime sleep episodes, n (%)	21 (30)	65 (38)	88 (34)	174 (35)	.46
Other, n (%)	0 (0)	1 (1)	4 (2)	5 (1)	.57^c^
No consequences, n (%)	1 (1)	1 (1)	3 (1)	5 (1)	.84^c^

a ISI: Insomnia Severity Index.

b Multiple choice question.

c Fisher exact test.

**Table 9.** Consequences of insomnia symptoms on daily life in EU5^a^ (n=730) participants with an ISI^b^ score >0**.** Participants were asked “What are/were the consequences of your sleep difficulty symptom(s) on your daily life during your most recent depressive episode?”

Consequences of insomnia symptoms^c^	EU5 participants with ISI score >0				
	Not currently in a depressive episode (n=87)	Unsure but experiencing depression symptoms (n=200)	Currently in a depressive episode (n=443)	Total (N=730)	*P* value
Irritability, n (%)	48 (55)	134 (67)	320 (72)	502 (69)	.006
Reduced productivity, n (%)	45 (52)	104 (52)	226 (51)	375 (51)	.97
Concentration difficulties, n (%)	40 (46)	113 (56)	272 (61)	425 (58)	.02
Increased caffeine consumption, n (%)	36 (41)	90 (45)	194 (44)	320 (44)	.85
Daytime sleep episodes, n (%)	25 (29)	49 (24)	97 (22)	171 (23	.35
Other, n (%)	0 (0)	3 (1)	5 (1)	8 (1)	.77^d^
No consequences, n (%)	5 (6)	14 (7)	19 (4)	38 (5)	.35

a EU5: France, Germany, Italy, Spain, and the United Kingdom.

b ISI: Insomnia Severity Index.

c Multiple choice question.

d Fisher exact test.

#### Impact of MDDIS Symptoms on Daily Life

When asked to rank the MDDIS symptoms that had the greatest impact on their lives, most participants in both regions identified depressed mood, lack of motivation, and tiredness as the most disruptive (Figure 5). Among those currently experiencing a depressive episode or symptoms, feelings of worthlessness, loss of energy, and diminished interest were also frequently prioritized.

### Perceived Relationship Between Insomnia Symptoms and Depression

**Figure 7.**
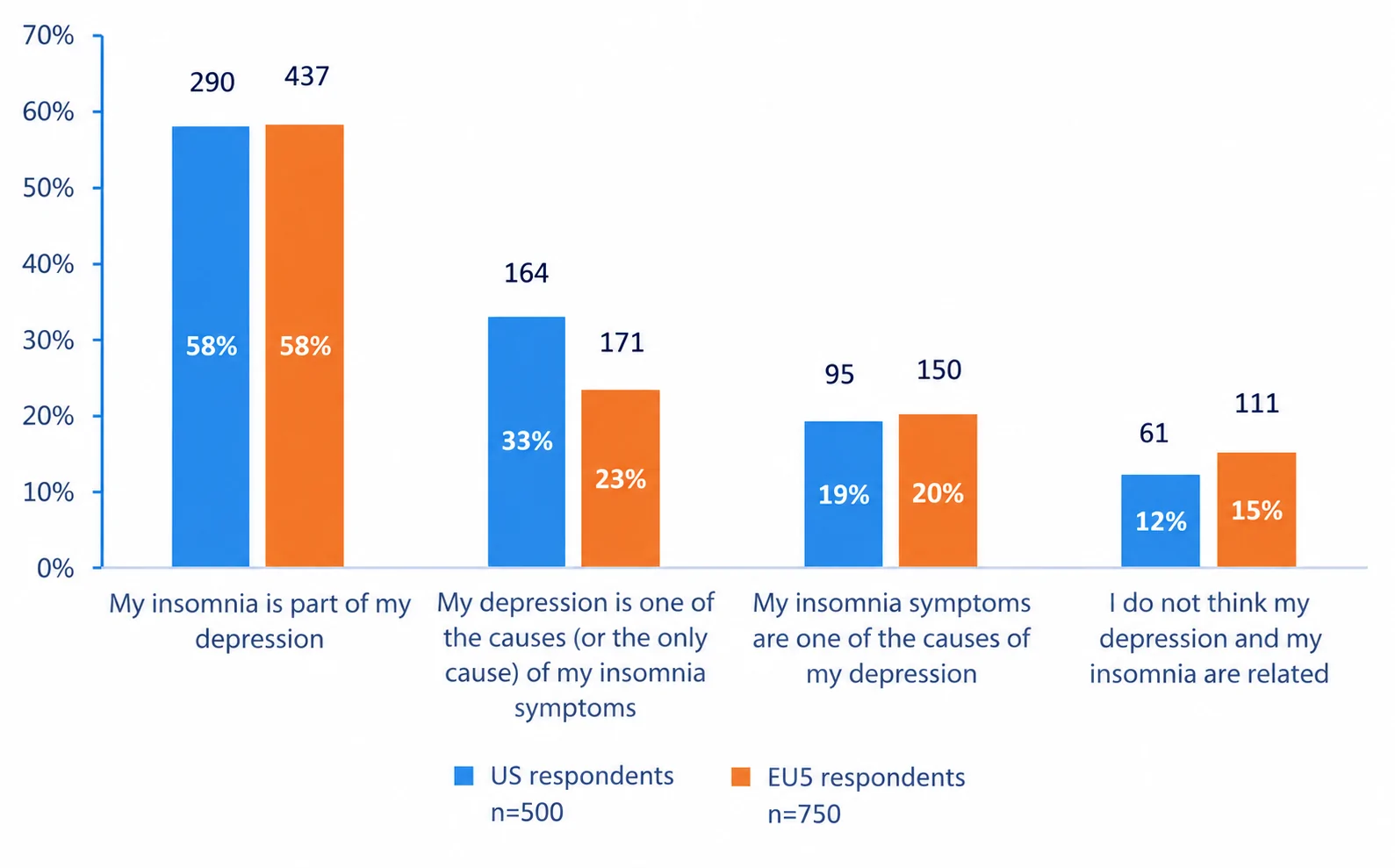
Perceived relationship between insomnia symptoms and depression in the US (n=500) and EU5 participants (n=750). EU5: France, Germany, Italy, Spain, and the United Kingdom.

When asked how they think insomnia is related to their depression, the majority of participants, 88% (439/500) in the United States and 85% (639/750) in the EU5, believed both conditions are linked. In both regions, 58% (United States: 290/500; EU5: 437/750) considered insomnia symptoms to be part of their depression, while 33% (164/500; United States) and 23% (171/750; EU5) believed their depression was a cause of their insomnia symptoms. Around 20% (United States: 95/500; EU5: 150/750) in each region felt that insomnia symptoms contributed to their depression (Figure 7).

### Satisfaction With Treatment

#### Patient Satisfaction With Antidepressant Treatment for Depressive Symptoms

Satisfaction with antidepressant effectiveness for depressive symptoms was mixed in both regions. In the United States, 41% (193/472) of participants were dissatisfied, 28% (135/472) were satisfied, and 31% (144/472) reported neutral satisfaction (Table S2 in Multimedia Appendix 2). The distribution of satisfaction ratings differed significantly according to current depressive episode status and depression severity (both *P*<.001) but not according to MDD duration; a greater proportion of patients currently in a depressive episode (121/245, 50%) or experiencing severe depression (93/159, 59%) reported being not satisfied or not satisfied at all. In the EU5 sample, 31% (197/647) of participants reported dissatisfaction, 35% (230/647) reported satisfaction, and 34% (220/647) reported neutral satisfaction. Similarly, satisfaction with antidepressants for depressive symptoms differed significantly according to current depressive episode status and depression severity (both *P*<.001), whereas no significant differences were observed across MDD duration categories. Overall, 40% (157/395) of those currently in a depressive episode and 46% (122/265) of those with severe depression reported being not satisfied or not satisfied at all.

#### Patient Satisfaction With Antidepressant Treatment for Sleep Symptoms

In the US sample, 57% (269/472) of participants were dissatisfied with the effectiveness of antidepressants for improving sleep, whereas 14% (68/472) reported being satisfied. Satisfaction with antidepressants for sleep symptoms differed significantly according to current depressive episode status (*P*=.01) and depression severity (*P*=.006); 35% (56/159) of participants with severe depression were not satisfied at all (Tables 10 and 11). In the EU5 sample, 41% (264/647) of participants reported being dissatisfied and 26% (168/647) reported being satisfied with the effectiveness of antidepressants for improving sleep. Similarly, satisfaction ratings differed significantly according to current depressive episode status and depression severity (both *P*<.001), with 20% (54/265) of those with severe depression reporting being not satisfied at all. In both regions, no significant differences were observed across MDD duration categories (Tables 10 and 11).

**Table 10.** Satisfaction with antidepressant effectiveness for sleep problems during most recent depressive episode.

Satisfaction with antidepressant effectiveness for sleep problems during most recent depressive episode	US population (n=472)					*P* values
	Not satisfied at all, n (%)	Not satisfied, n (%)	Neutral, n (%)	Satisfied, n (%)	Very satisfied, n (%)	
Overall	121 (26)	148 (31)	135 (29)	58 (12)	10 (2)	—^a^
MDD^b^ duration (years)						.62
0‐5 (n=184)	44 (24)	51 (28)	60 (33)	24 (13)	5 (3)	
6‐20 (n=146)	35 (24)	49 (34)	43 (29)	17 (12)	2 (1)	
>20 (n=142)	42 (30)	48 (34)	32 (23)	17 (12)	3 (2)	
Current depression status						.01
Not currently in a depressive episode (n=67)	12 (18)	13 (19)	24 (36)	15 (22)	3 (4)	
Unsure but experiencing depression symptoms (n=160)	39 (24)	51 (32)	45 (28)	23 (14)	2 (1)	
Currently in a depressive episode (n=245)	70 (29)	84 (34)	66 (27)	20 (8)	5 (2)	
Depression severity						.006^c^
None-minimal (n=13)	2 (15)	1 (8)	4 (31)	5 (38)	1 (8)	
Mild (n=63)	11 (17)	18 (29)	24 (38)	9 (14)	1 (2)	
Moderate (n=122)	22 (18)	38 (31)	39 (32)	19 (16)	4 (3)	
Moderately severe (n=115)	30 (26)	44 (38)	30 (26)	9 (8)	2 (2)	
Severe (n=159)	56 (35)	47 (30)	38 (24)	16 (10)	2 (1)	

a Not available.

b MDD: major depressive disorder.

c Fisher exact test.

**Table 11.** Satisfaction with antidepressant effectiveness for sleep problems during most recent depressive episode.

Satisfaction with antidepressant effectiveness for sleep problems	EU5^a^ population (n=647)					*P* values
	Not satisfied at all, n (%)	Not satisfied, n (%)	Neutral, n (%)	Satisfied, n (%)	Very satisfied, n (%)	
Overall	89 (14)	175 (27)	215 (33)	137 (21)	31 (5)	—^b^
MDD^c^ duration (years)						.19
0‐5 (n=278)	35 (13)	78 (28)	92 (33)	54 (19)	19 (7)	
6‐20 (n=213)	36 (17)	59 (28)	71 (33)	43 (20)	4 (2)	
>20 (n=156)	18 (12)	38 (24)	52 (33)	40 (26)	8 (5)	
Current depression status						<.001
Not currently in a depressive episode (n=84)	7 (8)	8 (10)	27 (32)	33 (39)	9 (11)	
Unsure but experiencing depression symptoms (n=168)	21 (13)	51 (30)	56 (33)	33 (20)	7 (4)	
Currently in a depressive episode (n=395)	61 (15)	116 (29)	132 (33)	71 (18)	15 (4)	
Depression severity						<.001^d^
None or minimal (n=24)	1 (4)	2 (8)	6 (25)	5 (21)	10 (42)	
Mild (n=57)	4 (7)	6 (11)	24 (42)	19 (33)	4 (7)	
Moderate (n=123)	7 (6)	29 (24)	41 (33)	37 (30)	9 (7)	
Moderately severe (n=178)	23 (13)	47 (26)	69 (39)	36 (20)	3 (2)	
Severe (n=265)	54 (20)	91 (34)	75 (28)	40 (15)	5 (2)	

a EU5: France, Germany, Italy, Spain, and the United Kingdom.

b Not available.

c MDD: major depressive disorder.

d Fisher exact test.

#### Unmet Needs and Desire for Alternative Treatments

**Figure 8.**
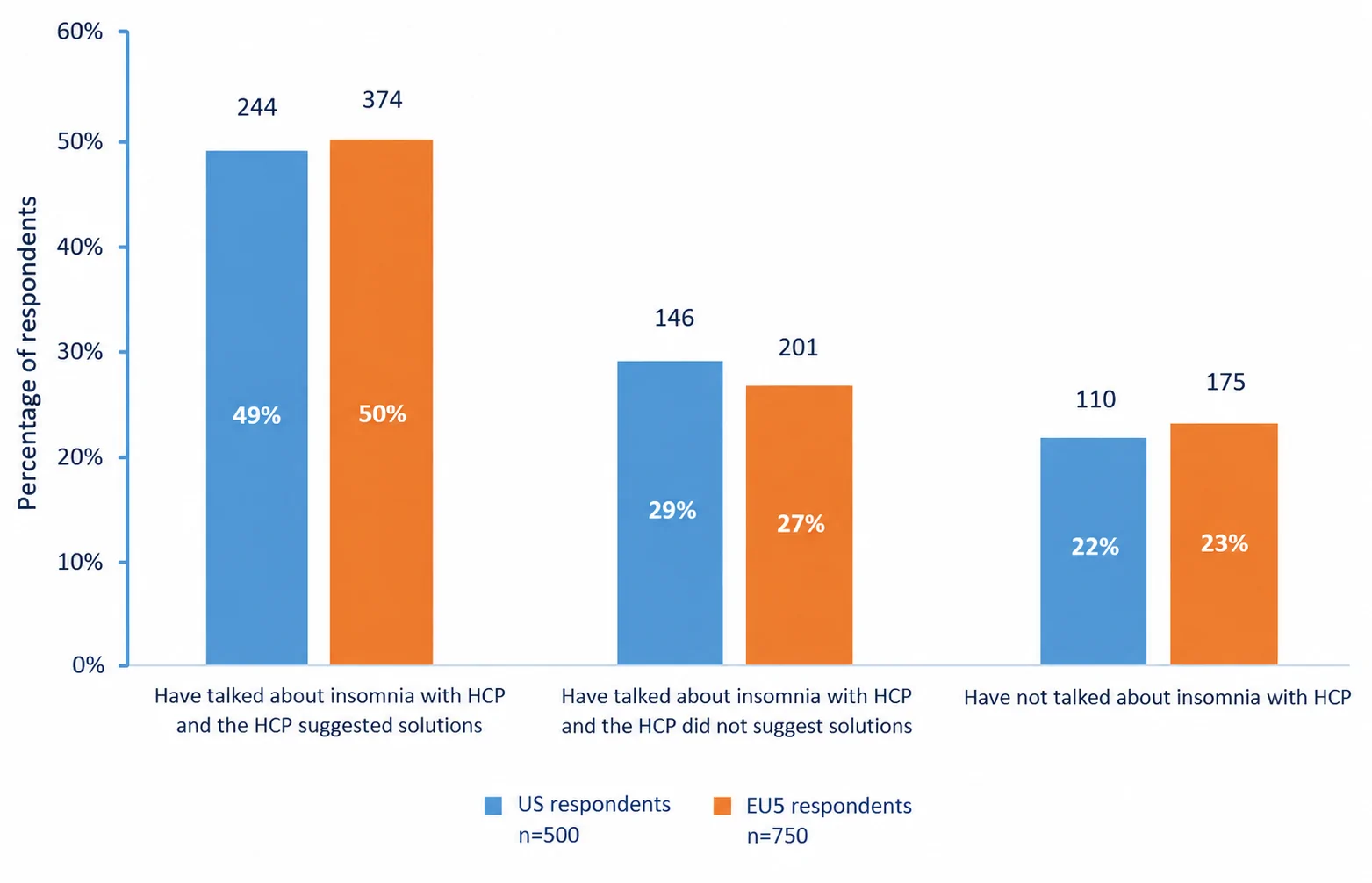
Discussion related to insomnia with health care professionals in the United States (n=500) and EU5 participants (n=750). EU5: France, Germany, Italy, Spain, and the United Kingdom; HCP: health care professional.

A strong desire for alternative treatments for both depression and sleep difficulties was reported (354/500, 71% in the United States and 488/750, 65% in the EU5), particularly among participants experiencing a current depressive episode or with more severe depression and insomnia symptoms. While most participants had discussed their insomnia symptoms with a clinician or another member of their care team (United States: 390/500 and 78%, EU5: 575/750, 77%), over one-third (United States: 146/390, 37% and EU5: 201/575, 35%) reported receiving no recommendations, especially those with more severe insomnia symptoms (Figure 8).

## Discussion

### Main Findings

This cross-sectional study provides patient-reported evidence for the burden of insomnia symptoms among individuals with MDD. Overall, insomnia symptoms were commonly reported despite treatment use, with moderate to severe symptoms more commonly reported among participants with more severe depression and those currently experiencing a depressive episode. Participants also described a substantial impact of insomnia symptoms on daily functioning, emotional well-being, cognitive functioning, and productivity, expressing low satisfaction with the perceived effectiveness of current treatments for sleep symptoms and a desire for additional therapeutic options. Collectively, these findings highlight the considerable patient-reported burden associated with insomnia symptoms and suggest that persistent sleep difficulties remain an important aspect of the patient experience.

This study shows that insomnia symptoms remain highly prevalent among patients with MDD, even among individuals receiving treatment. These findings are consistent with previous epidemiological studies showing that sleep disturbance is a common feature of depression and that insomnia symptoms may persist despite improvement in depressive symptoms, increasing the risk of relapse and recurrence [14,15,30,31]. Furthermore, the results suggest a link between insomnia severity and depression severity; participants with more severe depressive symptoms were more likely to report greater insomnia severity, in line with previous literature showing that short sleep and insomnia are independently associated with increased depression severity and symptom frequency [39,40]. These findings support the importance of considering sleep disturbance as a clinically relevant component of the overall burden of MDD, in particular among those with more severe depression.

Beyond their high prevalence, insomnia symptoms were perceived as having a substantial impact on multiple aspects of daily functioning. Participants reported impairments affecting emotional well-being, cognitive functioning, or work productivity, consistent with previous studies demonstrating that people with both depression and sleep problems experience daytime symptoms such as poor concentration and fatigue, and that insomnia symptoms have a strong negative impact on QoL and psychological distress [15,17].

Participants’ perceptions further illustrate the complexity of the relationship between insomnia symptoms and depression. Although insomnia is recognized as a core symptom of MDD [14], some participants still do not perceive their sleep difficulties as being part of their depression. This finding may reflect limited awareness of the relationship between these conditions and supports the need to provide patients with information regarding the coexistence of mood and sleep symptoms [14,16,30].

Treatment use was high, despite the prevalence of insomnia symptoms. The majority of participants reported dissatisfaction with the perceived effectiveness of antidepressants for improving sleep symptoms and expressed interest in alternative therapeutic options addressing both depression and sleep. Previous literature has observed that more than half of patients perceived antidepressants as insufficient for improving sleep and seek additional support [15]. However, this study did not assess treatment adherence, treatment duration, clinical appropriateness, or treatment resistance. Therefore, these findings should be interpreted as reflecting patients’ perceptions of persistent sleep difficulties despite treatment rather than evidence of inadequate clinical management.

Despite the well-established literature demonstrating an association between depression and insomnia symptoms [14,16,30-32,41], this association is not adequately reflected in current treatment practices. Although most participants reported discussing their sleep difficulties with a clinician or another member of their care team, over one-third indicated that no treatment suggestions were provided. As the study did not capture the reasons underlying these interactions, alternative explanations, including patient preferences, clinical judgment, or treatment prioritization, cannot be excluded. Nevertheless, these findings suggest a potential communication or care gap that warrants further investigation.

### Strengths and Limitations

A major strength of this study is its large multinational sample recruited through an online patient community, allowing the collection of patient-reported experiences from individuals with MDD across 6 countries [42]. The use of validated instruments (PHQ-9 and ISI) ensured standardized assessment of depression and insomnia severity, while the study-specific questionnaire provided valuable insights into patients’ treatment experiences, perceptions, and unmet needs. Compared with previous studies that primarily focused on the economic burden and health care resource use associated with insomnia symptoms in MDD [31,32], this study offers a broader understanding of the patient experience and highlights aspects of disease burden that are not routinely captured in clinical practice.

Several limitations should nevertheless be acknowledged. Recruitment through an online platform may have introduced selection bias by excluding individuals without internet access or those less familiar with digital tools, and by preferentially including patients who are more engaged in managing their condition. In addition, the sample was predominantly composed of cisgender women, which may limit the generalizability of the findings to the broader MDD population [7]. The observed differences in the proportion of participants identifying as transgender or nonbinary between the United States and the EU5 may also reflect cultural and societal differences in the willingness to disclose sex identity.

All data were self-reported, introducing the possibility of recall and reporting bias. In particular, the diagnosis of MDD was based on participant self-report and was not independently confirmed through medical records or health care professionals, which may have resulted in diagnostic misclassification. Furthermore, information regarding treatment adherence, treatment duration, treatment optimization, clinician rationale for treatment selection, and treatment resistance was not collected, limiting the interpretation of participants’ reported treatment experiences. Sleep duration, a factor known to influence the relationship between insomnia and depression, was also not assessed and may have acted as a confounder. Finally, the cross-sectional design precludes any conclusions regarding temporal or causal relationships between insomnia symptoms and depression severity.

Future longitudinal studies integrating both patient- and clinician-reported outcomes are warranted to better characterize the relationship between insomnia symptoms and the onset, course, and treatment response of MDD. Such studies should also investigate behavioral and educational aspects of insomnia management, including patients’ understanding of the relationship between sleep and depression, as well as strategies that promote adaptive coping and improve long-term outcomes.

### Implications for Clinical Practice

Given the well-established association between depression and insomnia symptoms reported in the literature [38], our findings further emphasize the importance of considering sleep symptoms during the clinical assessment of patients with MDD. Although this study cannot determine the appropriateness of current treatment strategies, the persistence of patient-reported sleep difficulties and the expressed need for additional therapeutic options suggest that insomnia symptoms remain an important component of patient care deserving continued clinical attention.

### Conclusion

This multinational patient study highlights the substantial burden associated with insomnia symptoms among individuals with MDD. Participants reported an impact of insomnia symptoms on daily functioning, emotional well-being, and productivity. More severe depression was accompanied by a greater proportion of participants with moderate or severe insomnia symptoms, and dissatisfaction with current treatment options was widespread. Around two-thirds of participants also expressed a desire for additional treatment options. Although the cross-sectional design and patient-reported nature of the data preclude conclusions regarding causality or the appropriateness of current clinical management, these findings suggest that insomnia symptoms remain an important component of the patient experience in MDD and identify a potential care gap that warrants further investigation.

## Supplementary material

10.2196/93231 Multimedia Appendix 1 Characteristics of EU5 study population per country.

10.2196/93231 Multimedia Appendix 2 Satisfaction with antidepressant effectiveness in treating depression during most recent depressive episode (US: n=472, EU5: n=647).

## Abbreviations

*DSM-5*: *Diagnostic and Statistical Manual of Mental Disorders* (Fifth Edition)
EU5: France, Germany, Italy, Spain, and the United Kingdom
GBD: Global Burden of Disease
ISI: Insomnia Severity Index
MDD: major depressive disorder
MDDIS: MDD experiencing insomnia symptoms
OTC: over-the-counter
PHQ-9: Patient Health Questionnaire-9
QoL: quality of life
