# Supplementary material for: Burden of Insomnia Among Individuals With Major Depressive Disorder: Cross-Sectional Observational Study
Source: Interact J Med Res. 2026 Sep 21;15:e93231. doi: 10.2196/93231 (PMC13592740; doi:10.2196/93231)
Supplement: Supplementary file 1 [file ijmr-v15-e93231-s001.pdf]

Table S 2: Satisfaction with antidepressant effectiveness in treating depression during most recent depressive episode (US: n=472, EU5: n=647)

Satisfaction with antidepressant effectiveness for depression	US population (N=472)						EU5 population (N=647)					
	Not satisfied at all	Not satisfied	Neutral	Satisfied	Very satisfied	P-value	Not satisfied at all	Not satisfied	Neutral	Satisfied	Very satisfied	P-value
Overall	65 (14%)	128 (27%)	144 (31%)	110 (23%)	25 (5%)		69 (11%)	128 (20%)	220 (34%)	189 (29%)	41 (6%)	
MDD duration						0.76						0.96
0-5 years	23 (13%)	45 (24%)	65 (35%)	42 (23%)	9 (5%)		33 (12%)	53 (19%)	96 (35%)	78 (28%)	18 (6%)	
6-20 years	22 (15%)	40 (27%)	41 (28%)	33 (23%)	10 (7%)		20 (9%)	40 (19%)	75 (35%)	66 (31%)	12 (6%)	
> 20 years	20 (14%)	43 (30%)	38 (27%)	35 (25%)	6 (4%)		16 (10%)	35 (22%)	49 (31%)	45 (29%)	11 (7%)	
Current depression status						<0.001						<0.001
Not currently in a depressive episode	3 (4%)	9 (13%)	19 (28%)	30 (45%)	6 (9%)		3 (4%)	3 (4%)	19 (23%)	37 (44%)	22 (26%)	
Unsure but experiencing depression symptoms	19 (12%)	41 (26%)	55 (34%)	38 (24%)	7 (4%)		11 (7%)	23 (14%)	70 (42%)	60 (36%)	4 (2%)	
Currently in a depressive episode	43 (18%)	78 (32%)	70 (29%)	42 (17%)	12 (5%)		55 (14%)	102 (26%)	131 (33%)	92 (23%)	15 (4%)	
Depression severity						<0.001						<0.001
None-minimal	0 (0%)	1 (8%)	1 (8%)	8 (62%)	3 (23%)		1 (4%)	0 (0%)	5 (21%)	7 (29%)	11 (46%)	
Mild	4 (6%)	7 (11%)	24 (38%)	25 (40%)	3 (5%)		3 (5%)	6 (11%)	18 (32%)	25 (44%)	5 (9%)	
Moderate	7 (6%)	27 (22%)	43 (35%)	35 (29%)	10 (8%)		4 (3%)	19 (15%)	38 (31%)	53 (43%)	9 (7%)	
Moderately severe	15 (13%)	39 (34%)	36 (31%)	23 (20%)	2 (2%)		14 (8%)	28 (16%)	68 (38%)	58 (33%)	10 (6%)	
Severe	39 (25%)	54 (34%)	40 (25%)	19 (12%)	7 (4%)		47 (18%)	75 (28%)	91 (34%)	46 (17%)	6 (2%)	

Row-wise percentage
