# Supplementary material for: Burden of Insomnia Among Individuals With Major Depressive Disorder: Cross-Sectional Observational Study
Source: Interact J Med Res. 2026 Sep 21;15:e93231. doi: 10.2196/93231 (PMC13592740; doi:10.2196/93231)
Supplement: Supplementary file 2 [file ijmr-v15-e93231-s002.pdf]

Table S 1: Characteristics of EU5 study population per country

	Germany N=154	Spain N=140	France N=167	Italy N=161	UK N=128	EU5 N=750
Age groups						
18-30 years old	38 (25%)	14 (10%)	34 (20%)	26 (16%)	11 (9%)	123 (16%)
31-60 years old	107 (69%)	107 (76%)	95 (57%)	101 (63%)	89 (70%)	499 (67%)
> 60 years old	9 (6%)	19 (14%)	38 (23%)	34 (21%)	28 (22%)	128 (17%)
HCP who diagnosed MDD, N (%)						
Psychiatrist	111 (72%)	95 (68%)	98 (59%)	127 (79%)	36 (28%)	467 (62%)
General practitioner	32 (21%)	36 (26%)	62 (37%)	20 (12%)	87 (68%)	237 (32%)
Others*	11 (7%)	9 (6%)	7 (4%)	14 (9%)	5 (4%)	46 (6%)
Severity of depression						
None-minimal	2 (1%)	3 (2%)	12 (7%)	5 (3%)	4 (3%)	26 (3%)
Mild	10 (7%)	8 (6%)	18 (11%)	22 (14%)	11 (9%)	69 (9%)
Moderate	32 (21%)	24 (17%)	31 (19%)	33 (20%)	26 (20%)	146 (19%)
Moderately severe	48 (31%)	33 (24%)	46 (27%)	43 (27%)	33 (26%)	203 (27%)
Severe	62 (40%)	72 (51%)	60 (36%)	58 (36%)	54 (42%)	306 (41%)
Severity of insomnia						
No clinical insomnia	13 (8%)	10 (7%)	20 (12%)	35 (22%)	7 (5%)	85 (11%)
Mild to moderate	42 (27%)	28 (20%)	52 (31%)	70 (43%)	34 (27%)	226 (30%)
Moderate	72 (47%)	65 (46%)	69 (41%)	45 (28%)	62 (48%)	313 (42%)
Severe	27 (18%)	37 (26%)	26 (16%)	11 (7%)	25 (20%)	126 (17%)

* Others include psychologist, therapist, mental health service, mental health counsellor, nurse practitioner, neurologist, counselling center, not specified
